# The why and how of sequential and combination therapy in osteoporosis. A review of the current evidence

**DOI:** 10.20945/2359-3997000000564

**Published:** 2022-11-10

**Authors:** Manju Chandran

**Affiliations:** 1 Singapore General Hospital Department of Endocrinology Osteoporosis and Bone Metabolism Unit Singapore Osteoporosis and Bone Metabolism Unit, Department of Endocrinology, Singapore General Hospital, Singapore

**Keywords:** Sequential, combination, anabolic, antiresorptives, osteoporosis, imminent fracture risk, drug holiday, sequence

## Abstract

It is now well recognized that over the lifetime of a patient with osteoporosis, more than one medication will be needed to treat the disease and to decrease fracture risk. Though current gaps in osteoporosis therapy can be potentially mitigated with sequential and combination regimens, how to move seamlessly amongst the multiple treatments currently available for osteoporosis for sustained efficacy is still unclear. Data from recent studies show that an anabolic agent such as teriparatide or romosozumab followed by an antiresorptive affords maximal gain in BMD and possibly better and earlier fracture risk reduction compared to a regimen which follows the opposite sequence. Sequentially moving to a bisphosphonate such as alendronate from an anabolic agent such as abaloparatide has also been shown to preserve the fracture reduction benefits seen with the latter. This sequence of an anabolic agent followed by an antiresorptive should especially be considered in the high-risk patient with imminent fracture risk to rapidly reduce the risk of subsequent fractures. The data surrounding optimum timing of initiation of bisphosphonate therapy following denosumab discontinuation is still unclear. Though data suggests that combining a bisphosphonate with teriparatide does not provide substantial BMD gains compared to monotherapy, the concomitant administration of denosumab with teriparatide has been shown to significantly increase areal BMD as well as to increase volumetric BMD and estimated bone strength. This narrative review explores the available evidence regarding the various sequential and combination therapy approaches and the potential role they could play in better managing osteoporosis.

## CASE SCENARIO

A 74-year old lady is referred for management of her osteoporotic fractures. Eight years ago, at age 66, while cleaning her house, she tripped over a table and hit her arm. At the emergency department she was found to have a right radius fracture, which was surgically fixed. No osteoporosis treatment was offered. At age 72, she slipped while walking and fell on her back. An x-ray showed grade 3 T11, T12 and L1 compression fractures of the 11th and 12th thoracic vertebrae and of the 1st lumbar vertebra. A DXA scan showed osteoporosis with lowest T-score of -3.2 at the lumbar spine. She was then started on alendronate. Now at age 74, she again has had another fall and has sustained a fracture of the left neck of femur. Repeat DXA scan showed worsening BMD with a significant change from the previous scan at both total hip and lumbar spine. Other than managing her frequent falls and ruling out secondary contributors to her osteoporosis, and optimizing her calcium and vitamin D intake, what is the best treatment option for her now?

## INTRODUCTION

**Figure 1:**
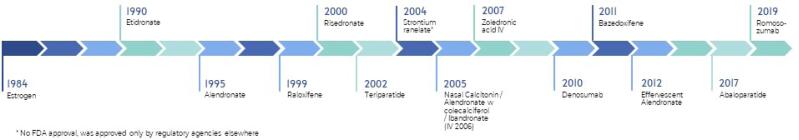
Pharmacological treatments for osteoporosis and year of FDA approval.

BMD decreases progressively with age and hence the incidence of osteoporosis and the risk of many fragility fractures increases in an exponential manner over the years of life of an individual. There is currently a plethora of medications available for the treatment of osteoporosis (Figure 1). On its own, each might be incapable of addressing all the pathophysiological perturbations that occur in osteoporosis. Additionally, the concerns over uncommon but serious side effects such as atypical fractures and osteonecrosis of the jaw, regulatory restrictions on duration of use of certain medications, the lack of further benefit when some anti-osteoporosis medications are used beyond a particular duration etc all make it necessary that over the life time of an individual, more than one anti-osteoporosis medication will need to be prescribed for many patients with the disease and who are at high risk for fracture. Several national and international guidance documents now advocate the use of medications in sequence to afford best protection for the patient with osteoporosis against fractures and those at very high risk (1–3). It is important thus that the effects of the available medications when used as sequential and combination therapy for osteoporosis be understood so that we can move seamlessly between the various treatment options we have for osteoporosis. This narrative review explores the available evidence underlying this relatively new concept and presents up-to-date practical guidance on the subject. While a full overview of all the studies undertaken on the subject is beyond the scope of this paper, this article will attempt to provide an overview of the currently available medications, how their individual mechanism of action affects their use and the impact on bone metabolism if they are discontinued. Key studies that illustrate the concept of sequential and/or combination therapy will be then highlighted.

## CURRENTLY AVAILABLE ANTI-OSTEOPOROSIS MEDICATIONS, THEIR MECHANISM OF ACTION AND EFFECTS OF DISCONTINUATION

### Antiresorptive therapies

#### Estrogen and selective estrogen receptor modulators (SERMS)

When used as pharmacologic agents, estrogen and SERMS act primarily as antiresorptive agents and suppress production of the osteoclast differentiation factor – receptor activator of nuclear factor kappa beta ligand (RANKL) – as well as proresorptive cytokines. They also promote osteoclast apoptosis and increase osteoblast production of osteoprotegerin (decoy receptor for RANKL) (4). Estrogen replacement has a consistent, favourable and large effect on bone density and reduces the risk of vertebral, nonvertebral and hip fractures (5,6). However its use is limited by adverse events including breast cancer, endometrial cancer, deep venous thrombosis and stroke (7,8), though it still offers a good risk-benefit profile when administered in the first 5-10 years after menopause (7). Rapid bone loss has been noticed to ensue after discontinuation of estrogen therapy and the reduction in hip fracture risk associated with it also progressively dissipates (9,10) though an increased fracture risk post discontinuation has not been demonstrated (11). SERMS have differential agonist and antagonist effects on estrogen receptors and therefore do not have the adverse effects on the breast and endometrium of estrogen (12). The two SERMS approved by the FDA in the treatment of osteoporosis – raloxifene and bazedoxifene – have proven efficacy in reducing bone remodelling, increasing bone density and in reducing vertebral fracture risk (13,14). Raloxifene's efficacy in reducing hip fracture risk is not proven in the main analyses of its pivotal trials and has only been shown in a post hoc analysis limited to patients with prevalent severe vertebral fractures at baseline (15). Similarly, bazedoxifene has shown nonvertebral fracture reduction only in a post hoc analysis of a subgroup of women at higher risk of fractures as assessed by FRAX in the Global Osteoporosis treatment study (16). Both are associated with DVT risk and raloxifene has been shown to be associated with a statistically significant increase in the risk of fatal stroke in post-menopausal women over the age of 60 years (17). Raloxifene is preferably suited for the younger post-menopausal women whose fracture risk is mainly vertebral. As the patient ages, it should preferably be substituted with alternate agents that have demonstrated efficacy in reducing hip and non-vertebral fractures. The antiresorptive effects of SERMS are rapidly reversible with an increase in bone remodelling and marked bone loss upon discontinuation (18) though an increase in fracture risk following stopping them has not been demonstrated so far.

#### Bisphosphonates

The nitrogen containing bisphosphonates – alendronate, risedronate, ibandronate and zoledronic acid – act by binding to hydroxyapatite and through inhibition of the enzyme farnesyl pyrophosphate synthase (FPPS), suppress protein geranylgeranylation and thus osteoclastic bone resorption (19). An inhibition of coupled bone formation also occurs. Initiation of treatment with bisphosphonates results in an initial closure of the remodelling space with resultant rapid increase in BMD. This is followed by a secondary heightened mineralization of the bone tissue and a much more modest BMD increase, with no further increase noted in hip BMD after 3-4 years of therapy. The bisphosphonates differ in the degree of binding to bone mineral with resultant variations in duration of retention in the skeletal matrix. Zoledronic acid has the highest binding affinity and risedronate the least. This persistent retention in bone leads to a long-term residual effect even when the agent is discontinued and has important implications when medication holidays and sequential therapy (discussed subsequently) are considered. The bisphosphonates also differ in their potency of inhibition of FPPS, with zoledronic acid being the most potent and alendronate being the least. Risedronate and ibandronate rank 2^nd^ and 3^rd^ respectively in order of potency (13). The bisphosphonates reduce vertebral, hip and non-vertebral fractures albeit with different efficacies in different populations (20,24). Ibandronate has not shown non-vertebral and hip fracture reduction except in a subpopulation with T-score of <-3 (25). Binding affinity plus the potency of inhibition of FPPS may both also explain differences in speed of onset of anti-fracture effect, offset of effect and whether there is an effect on non-vertebral sites. After treatment discontinuation, bone turnover markers remain lower than pre-treatment baseline for at least 2 years (26). The residual beneficial effects on fracture risk reduction has been suggested by extension studies of the Fracture Intervention Trial with oral alendronate (27) and the HORIZON Pivotal Fracture Trial with zoledronic acid (28) However, it must also be noted that recent evidence suggests that discontinuing bisphosphonates beyond 2 years may be associated with increased fracture risk (29).

#### Denosumab

Denosumab is the most rapidly acting and potent anti resorptive medication currently in use. It is a human monoclonal antibody that acts by reversibly blocking RANKL binding to the RANK receptor on osteoclasts and thereby markedly inhibiting osteoclastic recruitment, activity, and survival (30). Denosumab has been shown in multiple trials to increase BMD and to reduce vertebral, hip, and non- vertebral fracture risk. Unlike as with the bisphosphonates, denosumab use has been shown to be associated with persistent reduction of bone turnover and continued increases in bone mineral density (31) over 10 years and sustained reductions in fracture incidence (32–35). This effect may be due to modelling-based bone formation on the periosteal and endocortical surfaces being unaffected by denosumab (36). Denosumab has another advantage in that it may have superior effects on cortical bone as compared to alendronate and it may also reduce cortical porosity at the radius, tibia and the hip (37,38). Unlike with the bisphosphonates, the uptake of denosumab by the bone does not depend on bone turnover and values of bone turnover markers increase to above pre-treatment levels upon discontinuation. This is associated with bone mass loss within months after discontinuation and an increased incidence of multiple vertebral fractures (39). This characteristic has important implications since it necessitates follow-up with another anti-resorptive if therapy with denosumab needs to be discontinued for any reason especially if the patient has been on it for many years. It may also contribute to the exaggerated changes in bone turnover and significant bone loss that occur in patients transitioning from denosumab to recombinant human parathyroid hormone (PTH) analog therapy (40,42). The reason for this rebound phenomenon is still not clearly understood. Several postulations including upregulation of markers such as mRNAS for RANK and cathepsin K (involved in osteoclastogenesis and osteoclast activity), the activation of a pool of osteoclasts that were quiescent during the treatment period etc have been put forward by various investigators to explain this phenomenon (43).

**Anabolic therapies.** Mechanistic differences in action exist between the antiresorptive and anabolic agents (44). There are currently 3 approved anabolic therapies on the market. The PTH analogue- teriparatide, the parathyroid hormone related peptide (PTHrP) analogue-abaloparatide and the sclerostin inhibitor monoclonal antibody-romosozumab.

#### Teriparatide and abaloparatide

When administered intermittently as a once daily dosage, teriparatide and abaloparatide cause inhibition of osteocyte sclerostin expression, induction of osteoblastogenesis, and a decrease in osteoblast apoptosis (45). They also stimulate RANKL secretion by osteoblasts with resultant osteoclast activation and resultant bone resorption. Markers of bone formation are seen within days or weeks of initiating teriparatide. Though variations exist depending on the specific marker measured, bone formation marker levels usually peak within 1 year, and begin to decline thereafter, despite continued treatment (46). Increases in markers of bone resorption are delayed until after 1 month of initiation, but peak and subsequently decline during the second year of teriparatide. It has been shown through double fluorochrome labelled bone biopsy studies that teriparatide increases all types of bone formation (remodelling-based, modelling-based and overflow modelling-based) significantly in the cancellous and endocortical envelopes of bone with modelling based formation also increased in the periosteum (44). Since the phenomena of osteoblastogenesis and reduction in osteoblast apoptosis occur earlier and in a higher magnitude than the activation of osteoclasts, a net increase in trabecular bone mass and improvement of trabecular microarchitecture is seen (47). Though an increase in cortical porosity has been noted, the increase in cortical thickness due to periosteal apposition, the increased trabecular connectivity, and the transformation of trabecular rods to plates overall leads to an increase in bone strength (48–50). Though remodelling rates induced by teriparatide revert to baseline much before 24 months as evidenced by return of serum and urine markers of bone turnover to their pre-treatment baseline, the modelling-based bone formation continues to be stimulated (44,51). Teriparatide has shown conclusive vertebral and non-vertebral fracture reduction and has been demonstrated to increase spine, femoral neck and total body bone mineral density in the pivotal Fracture Prevention Trial (31). The study was terminated early before hip fracture reductions were seen. A subsequent study assessed fracture results in pooled data from 4 prospective observational teriparatide studies (52). This showed that teriparatide treatment was associated with significant decreases in hip fracture rate particularly for > 18 months of treatment, in real-world patients. The results however must be interpreted in the light of the non-controlled, observational design of the studies that were included.

It is unclear whether the pharmacological effects of PTH and PTHrP analogues differ based on the differential binding affinities to the two different PTH/PTHrP receptor configurations R^0^ and RG. In vitro and animal studies suggest that PTH and PTHrP analogies can differentiate between the two receptor conformations. It has been suggested that more efficient binding to R^0^ results in prolonged signalling and a greater calcemic response while more efficient RG binding is associated with a more transient response (53). This differential binding affinities of abaloparatide (selective binder to RG) and teriparatide (binding preferentially to R^0^) may thus account for some of the observed differences in bone resorption rates and hypercalcemia incidence (both less with abaloparatide) between the two agents though this is still not proven conclusively. Abaloparatide also has shown good vertebral and nonvertebral fracture (including wrist fracture) risk reductions (54–57). In the ACTIVE (Abaloparatide Comparator Trial in Vertebral Endpoints) Trial, abaloparatide was associated with modestly higher BMD gains especially at skeletal sites with cortical bone predominance and a slightly earlier and more efficacious fracture reduction than teriparatide (55). The authors conjectured that this was likely due to the lesser increases in bone resorption associated with abaloparatide. However, this finding must be interpreted with caution. A hierarchical sequencing approach was adopted for the statistical analysis in the trial which limits the comparison to at most exploratory. It is also difficult to accept that the 0.5%-1% higher BMD noted with abaloparatide as compared to teriparatide was due to differences in bone resorption marker levels between the two.

BMD increases (both areal BMD as measured by DXA as well as trabecular BMD measured by quantitative computerized tomography and fracture risk reductions while the patient is on teriparatide are mostly lost when the drug is discontinued (58,59). This again has important implications if these agents are used for treatment of osteoporosis since it necessitates that an alternative agent should be prescribed following cessation of their use.

#### Romosozumab

A humanized monoclonal antibody, romosozumab rapidly and transiently stimulates bone formation by inhibiting the effect of sclerostin thereby removing the inhibition by the latter molecule on proliferation, differentiation, and survival of osteoblasts (60). The inhibition of sclerostin also results in a more sustained decrease in bone resorption through reduction in RANKL synthesis (60). Thus, initially an increase in modelling based cortical bone formation is seen on the periosteum and endocortical surfaces (61,62). Subsequently, by about 12 months trabecular bone turnover is reduced.

The pivotal study with romosozumab was the FRAME, in which postmenopausal women with osteoporosis received romosozumab or placebo for a year followed by denosumab 60 mg subcutaneously once every 6 months for 12 months (63,64). It was shown that one year of romosozumab increased spine and hip BMD markedly and reduced vertebral and clinical fractures. The fracture risk reduction persisted even after transition to denosumab over 24 months. This data introduced the concept of the Foundation effect whereby rebuilding bone with the first one year of an anabolic agent such as romosozumab resulted in continued lower fracture risk after transitioning to an antiresorptive agent such as denosumab (63).

### The necessity for sequential and combination therapy in osteoporosis

Despite the development and availability of multiple antiosteoporosis medications, several dilemmas and strategy gaps in osteoporosis management exist. As mentioned earlier, increasing life spans necessitate that a patient with a chronic disease such as osteoporosis may have to take several medications sequentially over a long period of time. Whether we are truly adapting to the physiological needs of the skeleton with current therapies is unclear. Though antiresorptive agents decrease remodelling rates thereby attenuating the deficit in the mineralized bone matrix and prevent worsening of microarchitecture, the total bone matrix volume remains reduced as does the deterioration of microarchitecture that has already occurred. Patients at very high near-term or imminent risk of fracture (65) and those who have very low BMD need agents that are able to achieve significant increases in BMD and reduce fracture risk rapidly. This amount of rapid BMD increases that are needed may be difficult to achieve with antiresorptive medications but, are likely to be attained with an anabolic agent. It has been shown that BMD T-score improvements from baseline at the lumbar spine in patients treated with 1 year of romosozumab in FRAME are similar to that observed with 4.5 years of denosumab treatment in FREEDOM. One year of romosozumab followed by 1 year of denosumab treatment in FRAME led to BMD changes similar to 7 years of denosumab treatment (64). Though what constitutes “treatment failure” is still a point of debate (66), some patients may experience multiple fractures or a significant loss of BMD while on therapy despite being compliant and will need to switch therapies when this happens. It may also be necessary to employ sequential treatment to achieve a treatment target. Data from the FNIH Bone quality project show that the most important predictor of treatment mediated fracture risk reduction is an increase in hip T-scores (67). Post-hoc analysis of the FREEDOM Trial has suggested that fracture risk does not decrease further when total hip BMD T-scores increase beyond −1.5 (68). If the initial BMD T-score is −2 or above, it is highly possible that the above target will be achieved with a bisphosphonate. On the other hand, if total hip BMD is below −2, this attainment is unlikely with a bisphosphonate and therefore therapy might have to be initiated with a more potent medication or the bisphosphonate if already started may have to be replaced by a more potent one. Given that though an agent such as denosumab is likely to result in such a target being achieved and its discontinuation after years of therapy may result in rebound bone loss, it may be necessary to consolidate the gains achieved with it by sequentially following it up with a bisphosphonate.

### Sequential treatment

Possible treatment sequences include 1) an antiresorptive agent followed by an anabolic agent, 2) an anabolic agent followed by an antiresorptive agent and 3) an antiresorptive agent followed by another antiresorptive agent.

#### 1) Antiresorptive followed by an anabolic

Given the relatively higher costs of anabolic agents and because in most countries, reimbursement issues curtail their use unless there is documented treatment failure with one or more antiresorptive agents, as in the patient illustrated in the case example, most patients who are prescribed the former medications have likely had prolonged exposure to the latter ones. As mentioned earlier, bisphosphonates are embedded in the bone matrix and are likely to recirculate in this environment for several years. Prior bisphosphonate use potentially can influence the subsequent response to anabolics. Bisphosphonates especially those that are more potent and those that have longer skeletal half-lives have been shown to blunt the BMD responses that are normally induced by an anabolic such as teriparatide (69,70). Bisphosphonates are much more potent than raloxifene and therefore they cause more blunting than the latter medicine when used before an anabolic. This latter phenomenon was revealed in a prospective non-randomized study where after 18-36 months of treatment with alendronate or raloxifene, a switch to teriparatide was made. A reduction in hip BMD at 6 months was found in those previously treated with alendronate but not those in who had received raloxifene (71). Though this is concerning, it has been shown that vertebral and clinical fracture risk reduction seen with teriparatide treatment was not affected by previous bisphosphonate use or by interval between prior bisphosphonate use and initiation of teriparatide in the VERO study (72). It however must be noted that this study was powered to detect differences in vertebral fracture risk reduction between teriparatide and risedronate and not to detect differences between such subgroups (72).

In the DATA -SWITCH study which was an extension of the DATA trial, post-menopausal women who received teriparatide for 2 years after being on 2 years of denosumab experienced a transient decline in spine BMD that was manifest for 6 months after the switch, but had a longer and more profound decline in total hip and femoral neck BMD and a continued, progressive loss in distal radius BMD (40). This was accompanied by a significant increase in markers of bone turnover at 6 months. This particular sequence in which an anti-resorptive agent (denosumab) was followed by an anabolic agent (teriparatide) was also associated with reductions in total and cortical volumetric BMD on high-resolution peripheral quantitative computed tomography (HRpQCT) and reductions in cortical thickness and estimated strength by finite element analysis (FEA) (73). It is postulated that this pro-remodelling effect exerted by teriparatide is through the stimulation of osteoclast precursors that might have lain quiescent during denosumab therapy. Though the DATA-SWITCH study was not powered to detect fracture risk, it seems intuitive that this marked increase in bone remodelling and loss of hip BMD is likely to lead to a temporary increase in fracture risk. It is best therefore not to employ this treatment sequence in clinical practice. If a switch from denosumab to teriparatide is indicated, then it might be preferable to continue denosumab therapy with concomitant addition of teriparatide. The latter recommendation is derived from evidence from the DATA study (74) wherein combined teriparatide and denosumab had BMD gains more than that of teriparatide or denosumab monotherapy. This will be discussed later under the combination therapy section.

In the STRUCTURE study, postmenopausal women who had been previously treated with bisphosphonates for an average of 6 years were randomized to either romosozumab or teriparatide for a period of one year (71). Though BMD increases in both groups were less than that seen in treatment naïve women, it was more in women switched to romosozumab compared to those in women who were treated with teriparatide. A differential was noted in both cortical volumetric BMD at the hip as assessed by QCT and bone strength estimates on FEA with increases observed in the romosozumab group while decreases were noticed in the teriparatide group. However, it must be noted that the full effect of teriparatide at the hip is not seen until the end of 24 months and therefore these findings at one year could reflect an incomplete effect of teriparatide (75). In the romosozumab phase 2 dose-finding study and its extensions, women with low BMD (T-scores of ≤ −2.0 and ≥ −3.5) were randomized to multiple arms and interventions over a 6-year period (76). One of the analyses within this study focused on a small number of subjects (n = 16) who were randomized to placebo for 24 months, followed by denosumab for 12 months, and then received romosozumab for an additional 12 months. In women pre-treated with denosumab, net BMD gains with 1 year of romosozumab were lower than those in treatment-naïve women or those who had transitioned from alendronate. The kinetics of increase of the bone formation marker, PINP were also different with a slower, progressive elevation and delayed peak noticed. Some of the initial BMD gain noted with romosozumab might be related to overfilling of the remodelling cavities open at the time it is administered. The amount of bone remodelling surface is lower in patients who received prior antiresorptive agents compared to that in previously untreated patients particularly with prior denosumab (77). The delayed increase in serum PINP suggests that ambient bone remodelling activity at the time of romosozumab initiation is what is contributing to its early bone-forming effects. A progressive increase in β-CTX was also seen. This suggests that the antiresorptive potency of romosozumab is insufficient to completely prevent the bone turnover rebound that normally occurs after denosumab cessation. There was still however a positive net balance between resorption and formation, as shown by the small bone mass increase Thus for patients on denosumab who have had a suboptimal response or who must discontinue treatment for other reasons, switching to romosozumab may be a reasonable option since it improves spine BMD and maintains or produces a small increment in hip BMD. In contrast, in the DATA-Switch study, women who switched from denosumab to teriparatide had a rapid decline in hip BMD, and a large increase in serum β-CTX levels associated with both the effect of denosumab discontinuation and the pro-remodelling action of teriparatide (40). Further research is needed to confirm the BMD effects with romosozumab after longer denosumab therapy and to confirm that this treatment sequence will be protective against occurrence of multiple vertebral fractures associated with denosumab withdrawal. Although the transition from denosumab to romosozumab appears to be superior to the transition to teriparatide, it must be noted that this inference is being made based on results from two separate studies with a limited number of participants and with no fracture endpoints. Currently, there are no randomized controlled trials directly comparing these two regimens.

In summary, most studies suggest that anabolic therapies are likely to be more beneficial when administered to previously untreated patients. However, in real world practice, patients with severe osteoporosis are still likely to derive benefit from anabolics even if they are not treatment naïve and despite an element of blunting of the anabolic's effect with prior bisphosphonate therapy. Providing a treatment gap between antiresorptive therapy and subsequent anabolic treatment is unlikely to decrease this blunting. Therefore, it is not necessary to provide this gap.

#### 2) Anabolic followed by antiresorptive

As mentioned earlier, BMD increases, and fracture risk reductions obtained while the patient is on teriparatide are mostly lost when the drug is discontinued. It is thus necessary to administer an antiresorptive medication in follow-up.

In the European Study of Forsteo (EUROFORS), patients with severe postmenopausal osteoporosis who had been prescribed 1 year of teriparatide subsequently were randomized to receive either teriparatide or raloxifene or calcium with vitamin D (78). The sequence of raloxifene after teriparatide was able to maintain the gains in lumbar spine BMD that was obtained in the first year with teriparatide and resulted in significant further increases in hip BMD (78).

The results of the PaTH study confirmed that oral alendronate is clearly effective in preventing the bone loss that occurs post-discontinuation of PTH 1-84. Alendronate also caused a further increase in hip and neck of femur areal BMD as well as volumetric trabecular BMD at hip and lumbar spine (59). In the prospective European Forsteo Observational Study, 1,576 women with severe osteoporosis were treated with teriparatide for 18 months and then followed up for another 18 months. In those patients who received bisphosphonates after teriparatide, vertebral and non-vertebral fracture rates remained significantly low when compared to baseline between 0-6 months.

In the DATA-Switch study, a small number of postmenopausal women (n = 27) with osteoporosis who were originally randomized to receive teriparatide for 2 years were switched to another 2 years of denosumab (40). These patients were found to have a total 4 year increase in lumbar and hip BMD up to 18.3% and 6.6% respectively (40). It was also shown in a separate analysis of this study that switching from teriparatide to denosumab was associated with increases in the lumbar Trabecular Bone Score (an index of microarchitecture) and improvements in total and cortical vBMD, cortical thickness, and estimated strength on HRpQCT (41,73). Data from the ACTIVExtend and romosozumab trials shed light on whether fracture reduction is attained with the subsequent use of an antiresorptive following the use of an anabolic agent. In the ACTIVE Extension study (ACTIVExtend), women who received abaloparatide or placebo in the ACTIVE trial were transitioned to additional 2 years of open label oral alendronate. Patients in both abaloparatide and placebo groups were offered additional 2 years of open label oral ALN 70 mg weekly. At 24 month follow-up, there was an 84% relative risk reduction (RRR) in vertebral fracture, 39% RRR in nonvertebral fracture, a 34% RRR in all clinical fractures and a 50% RRR in major osteoporotic fractures in the abaloparatide to alendronate group compared to the Placebo to alendronate group suggesting that that alendronate therapy can preserve the fracture reduction benefits of abaloparatide (79). In the FRAME trial, persistent reduced vertebral and clinical fracture risk reduction was seen at 24 months after transition to denosumab from romosozumab (64). The ARCH study enrolled 4,093 postmenopausal women with osteoporosis and a fragility fracture who were then randomly assigned in a 1:1 ratio to receive monthly subcutaneous romosozumab or weekly oral alendronate in a blinded fashion for 12 months, followed by open label alendronate in both groups (80). Over a period of 24 months, not only was the BMD increase achieved during romosozumab therapy maintained, a 48% and 19% lower risk of new vertebral fractures and nonvertebral fractures respectively was observed in the romosozumab-to-alendronate group compared to the alendronate to-alendronate group. This represented a maintenance of risk reduction in vertebral fractures and further reduction in nonvertebral fractures with alendronate treatment (80). Though no fracture data are available, it has been shown in a recent phase 2 study that a single dose of 5 mg zoledronate maintains the robust BMD gains accrued with romosozumab (81). This may afford a convenient way to ensure that the skeletal benefits of romosozumab are maintained after its discontinuation.

It is thus clear from these studies that transitioning from an anabolic agent to an antiresorptive helps to maintain BMD and anti-fracture efficacy overall and in some instances improves them further.

### Cyclical treatment

As mentioned earlier, after 18-36 months of teriparatide, there is no evidence of ongoing stimulation of bone formation or remodeling, at least in cancellous bone (46). Thus, there appears to be a form of tolerance associated with daily administration over this time. Early direct stimulation of bone formation without prior resorption (i.e., modelling-based formation) might be more important to ultimate BMD accrual than later activation of bone remodeling. Repeated short cycles of TPTD can potentially dissociate the early modeling-based anabolic effect from the latter remodeling-based one and might surmount the tolerance that develops after 6-15 months of daily therapy. To evaluate this hypothesis, a randomized, open-label study in postmenopausal women with osteoporosis who were recruited concurrently into two parallel cohorts: those who were on alendronate for at least 1 year and those that were antiosteoporosis treatment naïve was conducted. Within each cohort, volunteers were randomized to daily teriparatide or teriparatide given for 3-months followed by 3 months off cycles for a total treatment period of 24 months (47,82). Cyclic teriparatide over 2 years was shown to improve BMD similarly to daily treatment in women who are on ongoing alendronate therapy, despite receiving only 50% of the usual teriparatide dose. However, there did not appear to be a BMD advantage to cyclic administration in treatment-naive women for up to 24 months.

#### 3) Antiresorptive followed by another antiresorptive

A position paper by the IOF had recommended that in the situation of treatment failure, a weak antiresorptive agent such as raloxifene or ibandronate be replaced with a stronger one such as alendronate or risedronate, and a strong orally administered antiresorptive agent be replaced by parenterally administered denosumab or zoledronic acid (66). It must be noted that these recommendations were made before most of the studies discussed earlier in this review were performed and before the introduction of the potent anabolic agent romosozumab.

The practice of switching from one antiresorptive to another is likely to continue for the following reasons 1) the data surrounding the possible lack of benefit and in some instances the frankly deleterious effect on BMD with the use of an anabolic agent sequentially after an antiresorptive agent, 2) the need to consolidate the gains in BMD and fracture risk reduction attained with denosumab after a desired target BMD is attained and/or to prevent the rebound bone loss associated with its discontinuation, and 3) due to reimbursement and cost issues associated with prescribing potent anabolics. It is therefore worthwhile at this juncture to review the evidence pertaining to this.

In a multicentre, randomized, study, 504 postmenopausal women with a BMD T-score of between −2.0 or below and −4.0 or more who had been receiving alendronate therapy for at least 6 months were either continued on the ALN or switched to denosumab 60 mg q monthly and were followed up for 1 year (83). It was noted that transition to denosumab produced greater increases in BMD at all measured skeletal sites and a greater reduction in bone turnover than did continued alendronate. No data on fractures were provided in this study (83). In another randomized, double blind trial, 643 postmenopausal women with osteoporosis who had been treated for at least 2 years but on average more than 6 years with oral bisphosphonates were randomized 1:1 to subcutaneous denosumab or intravenous zoledronic acid for 12 months (84). A greater inhibition of bone remodelling and greater BMD increases at all measured sites was seen in women transitioning to denosumab compared to those who were switched to zoledronic acid (84). On the other hand, the dilemma of what agent to switch to and the timing of its administration after denosumab withdrawal remains a perplexing one. The DAPS study was a 24-month open-label randomized crossover trial that included postmenopausal treatment naïve women who were randomized to receive either denosumab 60 mg once 6 monthly or alendronate 70 mg once weekly (85). Analysis that focused on patients who were switched to alendronate therapy after 12 months of denosumab indicate that 1 year of alendronate therapy can maintain the BMD gains achieved with 1 year of denosumab treatment. Interestingly women who lost BMD with alendronate in year 2 showed a greater percent change in BMD with denosumab in year one (85). This was attributed to the phenomenon of “regression to the mean” – a characteristic of imprecision of measurement. The investigators of the study postulated that in patients with high rates of remodelling, closure of the remodelling space with a potent yet reversible agent such as denosumab may have resulted in greater gains in BMD and that in these individuals, discontinuation of such a reversible agent may result in resumption of the same level of remodeling as before which may not be completely inhibited by a bisphosphonate (85). In a small case series, administration of zoledronic acid was shown to preserve a major part (73%-87%) of the gains in BMD obtained with denosumab 1 year after its discontinuation (86). These findings however conflict with another smaller case series showing only minimal efficacy of zoledronic acid administered 6-8 months after denosumab discontinuation to maintain BMD (87). A few studies have investigated treatment with zoledronic acid following short-term denosumab therapy for ≤ 2.5 years. In the DATA-HD Extension Trial, postmenopausal women were treated with a combination of teriparatide 40 mcg and denosumab for 9 months followed by 6 months of denosumab therapy before receiving a single infusion of ZOL, 5.5 to 8 months after the last denosumab. Twelve months after zoledronic acid, BMD was maintained at all sites; however, 27 months after the zoledronic acid injection, lumbar spine BMD had decreased (−3.7%) and bone turnover markers increased toward baseline levels (88). In a 2-year randomized study which investigated whether zoledronic acid has long-term efficacy in maintaining BMD after discontinuation of denosumab, the agent was administered 6 months or 9 months after the last denosumab or when bone turnover had increased (observation group). Zoledronic acid was readministered if cross-linked C-terminal telopeptide (CTX) levels increased ≥ 1.26 μg/L or BMD decreased ≥ 5% (89). This study found that a single infusion of ZOL given at any of three different time points after the last DMAB was not sufficient to maintain BMD or continuously suppress bone turnover markers during the first 12 months. During the second year of the study, BMD was maintained at all sites and CTX remained stable and within the premenopausal reference range in all treatment groups. It must be noted however that nearly half of the study population fulfilled the criteria for retreatment with zoledronic acid at some point during the study, with most of these patients belonging to the 6-month group. The investigators found that longer treatment duration with denosumab, and younger age were associated with greater likelihood of bone loss 24 months after the first injection of zoledronic acid (89).

Notwithstanding these data which still require clarity, it is recommended that if the person has been only on short-term treatment with denosumab up to ~ 2.5 years, then upon discontinuation alendronate can be given for 1-2 years or a single dose of zoledronic acid can be administered 6 months after the last denosumab injection. If on the other hand the person has been on denosumab for > 2.5 years, it is better to give a more potent bisphosphonate i.e., zoledronic acid 6 months after the last denosumab injection and to monitor the marker of bone resorption CTX at 3, 6 and 12 months and to readminister zoledronic acid if CTX values are above the premenopausal range. If CTX levels are unable to be measured, the recommendation is to repeat the zoledronic acid infusion in 6 months.

**Figure 2:**
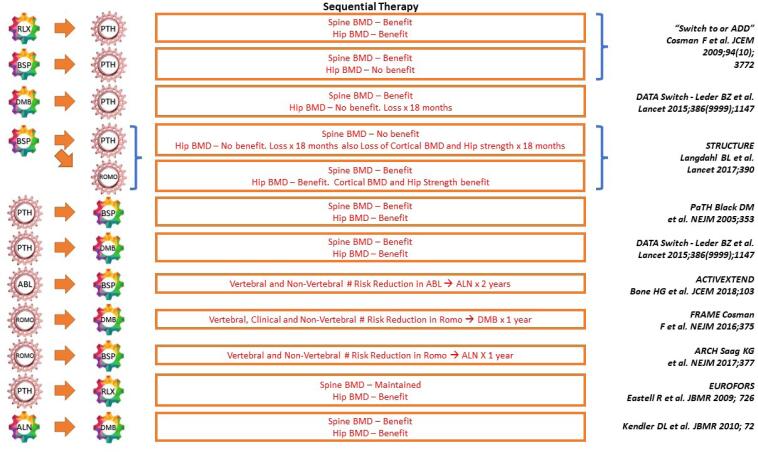
Sequential therapy.

A summary of some of the studies illustrating sequential therapy is provided in Figure 2.

### Combination therapy

Several therapy combinations are possible in osteoporosis. Most studies that have explored this option have been small ones evaluating bone turnover markers or BMD as outcomes. Combining two strong antiresorptive agents has not shown any benefit, however, given that anabolic agents such as teriparatide and abaloparatide secondarily stimulate bone resorption, it is instinctive to think that combining an antiresorptive agent with an anabolic would improve the efficacy of the latter.

**Figure 3:**
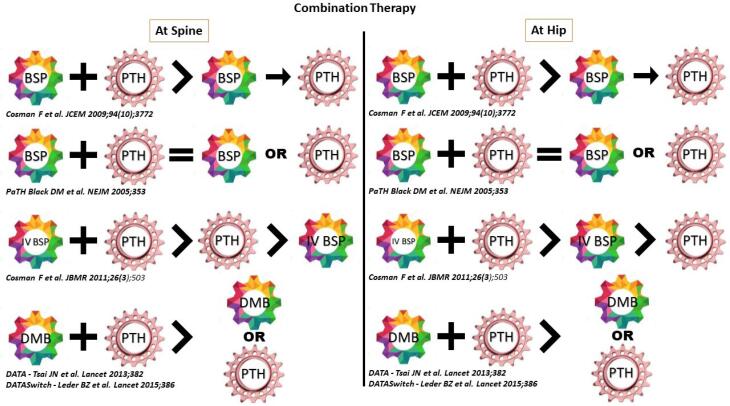
Combination therapy.

BSP: Bisphosphonate; PTH: Parathyroid Hormone; IV: Intravenous; DMB: Denosumab, >: More than; =: Same as

In a “Switch to or Add” study, a randomized open label trial, subjects who had been on alendronate or raloxifene for mean durations of 42.1 months, and 43.1 months respectively were switched to or had teriparatide added to their existing anti osteoporosis regimen (90). The bone density responses were greater in the Add vs. Switch patients in both the alendronate stratum and the raloxifene stratum though the BMD gains from adding teriparatide to a weaker antiresorptive such as raloxifene appeared to be slightly higher than gains from adding it to alendronate. In the PaTH trial, the effect of combining PTH 1-84 with alendronate was compared with monotherapy of either treatment alone (91). Though the bone mineral density at the spine increased in all the treatment groups, there was no significant difference in the increase between the PTH1-84 group and the combination therapy group. The volumetric density of the trabecular bone at the spine increased substantially in all groups, but the increase in the PTH1-84 group was about twice that found in either of the other groups. Bone formation increased markedly in the PTH group but not in the combination therapy group. Taken together, these results suggest that concurrent use of alendronate may reduce the anabolic effects of PTH and that there is no beneficial effect of co-administration of PTH analogues and frequently administered bisphosphonates. The potential of combining PTH analogues with a less frequently administered but more potent bisphosphonate was explored in a trial in which a single infusion of zoledronic acid in combination with teriparatide 20 mcg daily was compared against monotherapy with either drug for 1 year (92). An increase in lumbar spine BMD that was similar in the combination and teriparatide groups and higher than in the zoledronic acid alone group, and an increase in hip BMD that was similar in the combination and zoledronic acid groups and higher than in the teriparatide alone group was seen. This suggests that a single administration of zoledronic acid does not deleteriously impact the anabolic response of teriparatide at sites with high trabecular content such as the lumbar spine, but concurrently and possibly beneficially prevents the stimulation of cortical remodelling seen with teriparatide at the hip. The most promising combination tested to date is the concomitant use of teriparatide and denosumab. The DATA trial compared the effects of combining teriparatide and denosumab to monotherapy with either for 2 years (93). Combined treatment increased BMD more than either treatment alone. Most of the benefit of combination therapy was apparent in the first 12 months of treatment during which spine BMD increased by over 9% in the combo group vs approximately 6% in the TPT or DMB groups and total hip BMD increased by ~ 5% in the combo group compared to < 1% and 2% in the TPT and DMB groups. Additionally radius and tibia HRpQCT assessed volumetric BMD, cortical thickness and estimated bone strength increased more in women treated with the combination compared to teriparatide or denosumab alone while cortical porosity which progressively increased in women treated with teriparatide alone over the 24 months remained stable in women treated with the combination (41). However, interestingly, bone resorption markers in the combination group were similar to the markers in the denosumab alone group, whereas markers of bone formation were more suppressed in those treated with denosumab alone than in those receiving combo. This suggested that though the combination may blunt partly the anabolic effect of teriparatide, the overall beneficial effect of combined denosumab and teriparatide on BMD may be related to denosumab's ability to fully block the pro resorptive effects of teriparatide while still allowing teriparatide induced stimulation of modelling-based bone formation. This is unlike as when a bisphosphonate is combined with teriparatide, where teriparatide is still able to stimulate bone resorption even in the presence of the antiresorptive drug. Further support to combination therapy affording larger gains in BMD than monotherapy has been provided by the DATA HD study in which combined treatment with a higher dose of daily teriparatide (40 mcg) and 6-monthly denosumab increased spine and hip BMD even more than standard combination therapy (94). A summary of some of the studies illustrating combination therapy is provided in Figure 3.

**Cautions and caveats:** While the beneficial effects of sequential therapy especially one in which an anabolic is followed by an antiresorptive are clear, certain caveats should be considered. A regimen in which a moderately potent antiresorptive is followed by a stronger one has the potential to be associated with a higher risk of adverse events such as atypical fractures and osteonecrosis of the jaw. Though such an imbalance was not seen the FRAME study (64) in which romosozumab therapy was followed by denosumab, in the ARCH trial, adjudicated major cardiovascular events were more frequent in the romosozumab group than in the alendronate group during the double-blind period (80).

Cost issues must be taken into consideration. A few studies have explored the cost-effectiveness of sequential regimens. In a study from China, sequential denosumab/zoledronic acid was found to be more cost-effective than zoledronic acid monotherapy at older ages starting from 70 years old (95). Anabolic treatments are unlikely to be cost-effective when modelled on their own and payers often require documentation of use of antiresorptive therapy prior to anabolics. A recent analysis suggested that about 25%-30% favourable outcomes (in terms of fractures prevented and QALYs gained) could be obtained by initiating sequential treatment with abaloparatide compared to one beginning with alendronate (96). The abaloparatide/alendronate sequence was cost-effective compared to alendronate monotherapy in women ≥ 60 years of age at high-risk for fractures (96). Another study conducted in Sweden in 2021 indicated that treatment with romosozumab followed by alendronate can be a cost-effective treatment option for postmenopausal women with severe osteoporosis at high risk of fracture, with the romosozumab to alendronate sequence showing a 97.9% probability of being cost-effective against alendronate alone (97). These findings could be important to support decision makers for the optimal use of anabolic and antiresorptive treatment.

In conclusion, an expansion of the armamentarium of antiosteoporosis medicines has made it possible to treat the disease effectively and safely. Emerging evidence suggest that therapy should be initiated with an anabolic agent in patients who are at high risk to attain BMD gains quickly. Bisphosphonates are the only medications for which a drug holiday may be considered after an appropriate duration of use. Anabolic therapy after a potent antiresorptive such as alendronic acid is associated with an initial blunting in BMD response, however, reassuringly this does not seem to result in increased fracture risk. The transition from denosumab to teriparatide should be avoided to prevent accelerated bone turnover and bone loss. After denosumab is discontinued, the optimal timing of zoledronic acid administration remains unclear though available studies suggest that administering it 6 months after the last dose of denosumab may at least partially rectify the bone loss associated with the latter agent's discontinuation. The effects of all currently available anabolic agents appear to be reversible and therefore the administration of an antiresorptive medication is needed after their discontinuation. There is currently no fracture data to support combining an anabolic and antiresorptive. However, the gains seen in BMD by combining teriparatide and denosumab in the DATA and DATA-HD studies suggests that this synergistic effect may be employed to improve skeletal integrity in osteoporosis.
